# Public Health Round-up

**DOI:** 10.2471/BLT.18.010918

**Published:** 2018-09-01

**New evidence on breastfeeding**

The World Health Organization (WHO) and the United Nations Children’s Fund (UNICEF) have published a new report showing that three in five babies worldwide are not breastfed within the first hour of life. WHO recommends that babies are exclusively breastfed until they are six months old. Thereafter, babies should be given nutritious complementary foods and continue breastfeeding up to the age of two years or beyond.

**Figure:**
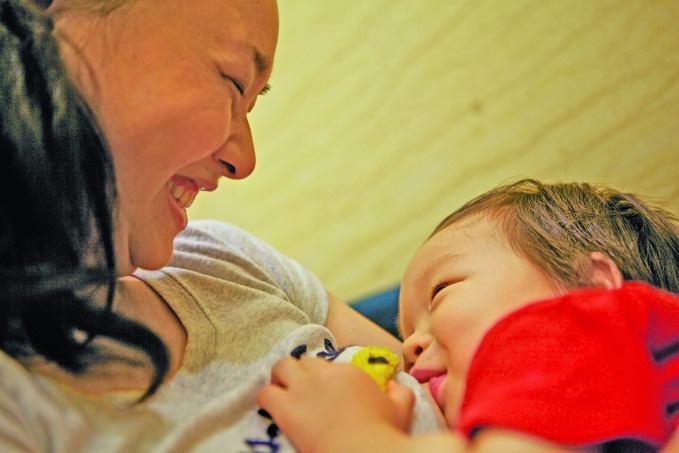
© UNICEF/Jerry Liu

## Ebola outbreak

The World Health Organization (WHO) and its partners are working with the government of the Democratic Republic of the Congo in response to a new outbreak of Ebola virus disease. This outbreak has now been declared a Grade 3 Emergency as per WHO's Emergency Response Framework.

It is the country’s 10th Ebola outbreak, but the first such outbreak in a densely populated active conflict zone, North Kivu province.

North Kivu hosts over 1 million displaced people and shares borders with Rwanda and Uganda, where there is a great deal of cross border movement and trade.

On those borders, 28 key points of entry have been identified and surveillance stations, to detect any new Ebola and viral haemorrhagic fever cases, were put in place. WHO is also working with neighbouring countries to ensure that the health authorities are prepared to detect cases.

“All of those participating in the response must be able to move freely and safely in conflict areas to do the work that is needed to bring the outbreak under control,” said WHO Director-General Tedros Adhanom Ghebreyesus, when he visited the country last month to support the response.

WHO deployed a mobile laboratory to provide on-the-spot testing for Ebola to the city of Beni on 2 August.

As of 15 August, more than 100 WHO staff had arrived in the affected areas.

Treatment centres have been set up by the nongovernmental organizations Alima and Médecins Sans Frontières France in Beni and Mangina, respectively.

With support from WHO, the government of the Democratic Republic of the Congo launched an Ebola vaccination campaign for high-risk populations in North Kivu Province on 8 August. Thousands of doses of the rVSV-ZEBOV Ebola vaccine are available in the country, with more ready to be delivered if needed.

Safe burials have been carried out in Beni with systems being put in place by the International Federation for the Red Cross and Red Crescent and partners supporting the health ministry to ensure that safe and dignified burials will be conducted throughout the affected zones.

http://bit.ly/2MKIay9

## Breastfeeding within an hour after birth

Three in five babies are not breastfed within the first hour of life, increasing their risk of death and disease, according to a new report.

The report, entitled *Capture the moment - early initiation of breastfeeding: the best start for every newborn, *published**by the United Nations Children’s Fund (UNICEF) and WHO, analyses data from 76 countries.

Despite the importance of early initiation of breastfeeding, many newborns wait longer than one hour, because they are separated from their mothers and/or rehydrated with water, cows’ milk or reconstituted breast-milk substitutes. Often, this happens when babies are delivered by caesarean section and because of other gaps in the quality of care provided to mothers and newborns.

Studies, cited in the report, show that newborns who began breastfeeding between two and 23 hours after birth had a 33% greater risk of dying compared with those who began breastfeeding within one hour of birth.

Among the newborns that started breastfeeding a day or more after birth, the risk was more than twice as high.

The report urges governments, donors and other decision-makers to adopt strong legal measures to restrict the marketing of infant formula and other breastmilk substitutes.

http://bit.ly/2MpDOQ4

## Tackling antimicrobial resistance

Countries are making significant steps in tackling antimicrobial resistance (AMR), but serious gaps remain and require urgent action, according to a new report.

The report, entitled *Monitoring global progress on antimicrobial resistance,* was released on 24 July by the Food and Agriculture Organization of the United Nations, World Organization for Animal Health (OIE) and WHO.

“Supporting low- and middle-income countries to follow guidance of responsible and prudent use of antimicrobials in animals is an urgent priority,” says Dr Matthew Stone, OIE Deputy Director-General.

Promising findings include 105 countries with a surveillance system in place for reporting drug-resistant infections in human health and 68 countries with a system for tracking consumption of antimicrobials. 93 countries reported having a multisectoral action plan to address AMR and 79 countries have a functioning co-ordination mechanism for AMR.

In addition, 123 countries reported that they have policies to regulate the sale of antimicrobials, including the requirement of a prescription for human use – a key measure to tackle overuse and misuse of antimicrobials.

A total of 67 countries report they have legislation in place to control aspects of production, licensing and distribution of antimicrobials for use in animals. However the remaining 56 of the 123 countries either said that they had no national policy or legislation, or that they were unable to report whether they had such policies in place.

The report charts progress in 154 countries and reveals wide discrepancies.

Some, including many European countries, have been working on AMR policies in human and animal sectors for more than four decades.

http://bit.ly/2BbPwtr

## Cholera vaccine in Yemen

Thanks to the efforts of more than 3000 local health workers, WHO, UNICEF and partners carried out an oral cholera vaccination campaign in Yemen’s Hudaydah and Ibb governorates last month. 

In total almost 400 000 people were reached with vaccine in the districts of Al-Hali and Al-Marawia in Hudaydah and the district of Hazm Al-Udain in Ibb governorate. There have been more than 1 million cases of suspected cholera with 2316 deaths in Yemen since April 2017. 

## Health needs in the West Bank and Gaza

WHO has provided life-saving medicines and medical supplies to hospitals and frontline trauma stabilization points last month, amid increasing violence in the West Bank and Gaza.

As of 11 August 164 people have been killed and 17 566 injured since the start of the demonstrations on 30 March.

Three health workers have been killed and 373 health workers have been injured.

Trauma stabilization points have been established throughout critical zones of the West Bank and Gaza in order to respond to the mass influx of casualties of the ongoing violence.

At these points, people with minor injuries are treated, and those with major injuries are stabilized and referred for definitive care. 

As of 15 August, 500 000 emergency and trauma patients have been treated with supplies from WHO’s recent delivery of medicines, assistive devices and medical equipment.

Of the 8695 people hospitalized to date, almost 50% suffer from gunshot wounds. Almost 20% of those being admitted to hospital are children. Psychosocial support is being provided through six WHO-supported mental health teams. Of the health workers, three were killed and 26 injured by live ammunition, a further 40 health workers were affected by tear gas and 13 were hit with shrapnel. A health-care centre for people with disabilities has been damaged, as well as at least 68 ambulances.

“The high numbers of trauma casualties, particularly with complex limb injuries, is continuing to increase,” said Gerald Rockenschaub, head of WHO’s office for the West Bank and Gaza.

http://bit.ly/2P5kSEO

**Cover photo**

Fishermen poling a skipjack tuna during the January 2018 Oceanic Fisheries Programme where the Pacific Community completed a successful tuna tagging voyage, releasing almost 28,000 tagged tuna into the Pacific Ocean.  Tuna fisheries in the Pacific Ocean employ millions of people and provide sustenance to millions more, but threats such as overfishing and climate change put this vital natural resource at risk.

**Figure:**
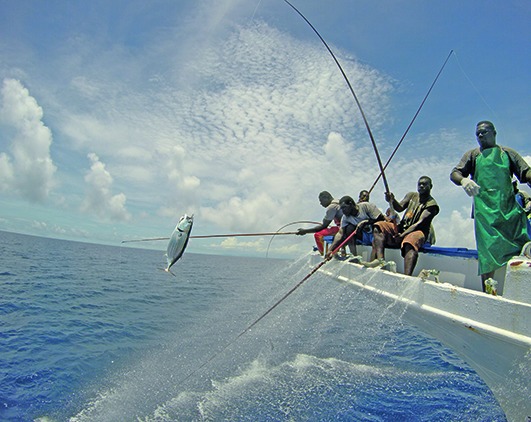
(Pacific Community)

## Measles prevention

WHO’s Regional Office for Europe and the National Public Health Agency of the Republic of Moldova recently convened paediatricians, infectious disease specialists and epidemiologists for measles prevention, surveillance and control training in the city of Tiraspol on the left bank of the Dniestr.

Participants analysed the response to recent measles outbreaks in the country’s northern Transdnistria region. Experts from the National Public Health Agency and the Nicolae Testemitanu Department of Infectious Diseases of the State University of Medicine and Pharmacy in the Moldovan capital Chisinau, delivered presentations and facilitated technical discussions.

The training was financially supported by the Swiss Agency for Development and Cooperation through the Multi-Partner Trust Fund and is being coordinated by the WHO Country Office in Chisinau.

This year, the country has reported some 37 measles cases to WHO, including some in the Transnistria region. The first cases were imported from neighbouring Romania and Ukraine.

More than 30 000 cases of measles were reported in WHO’s European Region in the first six months of 2018.

http://bit.ly/2wbtVLY

**Looking ahead:**

**26 September – UN General Assembly High-Level Meeting on the fight against tuberculosis, New York**

**27 September – Third UN High-level meeting on NCDs, New York **

**1- 4 October – Healthy Cities conference, Belfast **

**25-27 October – General Conference on Primary Health Care, Astana **

**30 October - 1 November – WHO’s First Global Conference on Air Pollution, Geneva**

